# A Narrative Review Exploring the Similarities between Cilento and the Already Defined “Blue Zones” in Terms of Environment, Nutrition, and Lifestyle: Can Cilento Be Considered an Undefined “Blue Zone”?

**DOI:** 10.3390/nu16050729

**Published:** 2024-03-02

**Authors:** Silvana Mirella Aliberti, Antonio Donato, Richard H. W. Funk, Mario Capunzo

**Affiliations:** 1 Department of Medicine, Surgery and Dentistry “Scuola Medica Salernitana”, University of Salerno, 84081 Salerno, Italy; 2 Institute of Anatomy, Technische Universität Dresden, 01307 Dresden, Germany; 3 Division of Preventive Medicine, Dresden International University (DIU), 01067 Dresden, Germany; 4 Complex Operational Unit Health Hygiene, University Hospital “San Giovanni di Dio e Ruggi d’Aragona”, 84131 Salerno, Italy

**Keywords:** Cilento, Blue Zones, similarities, environment, nutrition, lifestyle

## Abstract

Longevity is rightly considered one of the greatest achievements of modern society, not only as a function of lifespan, but, more importantly, as a function of healthspan. There are Longevity Blue Zones (LBZs), regions around the world, such as in Okinawa, Japan; the Nicoya Peninsula, Costa Rica; Loma Linda, California; Icaria, Greece; and Ogliastra, Sardinia, that are characterized by a significant percentage of residents who live exceptionally long lives, often avoiding age-related disability to a significantly higher degree than in the Western way of life. Longevity is not a universal phenomenon, so if there are places in the world with characteristics similar to the LBZs, it is important to identify them in order to better understand what other factors, in addition to the known ones, might contribute to a long and healthy life. This narrative review aims to identify common factors between Cilento and the five LBZs, taking into account environmental, nutritional, and lifestyle factors. Articles from 2004 to the present, limited to studies published in English, German, and Italian, were searched in PubMed/Medline, Scopus, and Google Scholar. The co-authors agreed on 18 final reference texts. In order to evaluate the similarities between Cilento and the LBZs, a descriptive comparative approach was used. Cilento and the LBZs share several common factors, including a hilly altitude ranging from 355 to 600 m; a mild climate throughout the year, with temperatures between 17.4 and 23.5 degrees Celsius; traditional professions, such as agriculture and animal husbandry; and a predominantly Mediterranean or plant-based diet, with typical recipes based on legumes, tubers, vegetables, and extra virgin olive oil. Additionally, maintenance of strong intergenerational family relationships, religious devotion, and social relationships within the community are also prevalent. Given the similarities to Cilento, one might wonder if this is an LBZ waiting to be discovered. The lessons learned from this discovery could be applied to the general population to protect them from non-communicable chronic diseases and help slow the aging process.

## 1. Introduction

The proportion of older people in the world’s population is growing. Their increase is due to the aging of the baby boom generation, declining birth rates, and the significant decline in age-related mortality since 1950 [1,2,3], so that there are now 727 million older people (aged 65 and over) and half a million centenarians (aged 100 and over) in the world [4]. By 2050, the 65+ population is expected to double to over 1.6 billion [4].

Under this scenario, maintaining vigor, resilience, and acceptable levels of health and functional autonomy across the lifespan is a major challenge and a research priority for individual well-being and for ensuring the economic sustainability of healthcare systems. Longevity is rightly regarded as one of the greatest achievements of modern society, not only as a function of lifespan, but, more importantly, as a function of healthspan [5]. Longevity is measured by age, and a person who is at least eighty-five years old is usually considered to have a long life. Each year lived at a very old age is significantly more extraordinary than the previous one, because the extreme limits of the human lifespan have been reached. Centenarians are individuals who have reached an exceptionally advanced age [2,6]. Centenarian status is sufficiently rare yet convenient for case–control studies because it is an easily understood and well accepted criterion of longevity. According to a number of studies, these individuals have a lower incidence of chronic diseases [7,8], a lower incidence of morbidity [9,10], and a longer healthy lifespan [11,12]. Thus, several studies in countries around the world, including Italy [5,13,14], Germany [15], Japan, and the United States [16], made the list of centenarians. Because the number of people who survive to old age is small, many prospective cohort studies have focused on reaching less extreme ages, such as 85, 90, or 95 [11]. Scientific studies in long-lived populations are important because they allow us to understand what factors can modify the epigenome over a lifetime and have beneficial effects on health [17,18,19]. Over the past two decades, research has sought to elucidate the factors that contribute to longevity and whether there are lessons that can be applied to the general population. Many studies have linked genetic [20,21,22], macroenvironmental [5,14,23], and microenvironmental [24,25,26] factors to longevity in humans.

The discovery of Longevity Blue Zones (LBZs), geographic areas characterized by a significant proportion of residents who live exceptionally long lives and often escape age-related disability, has captured the interest of scientists. These areas include residents of Okinawa, Japan; the Nicoya Peninsula, Costa Rica; Loma Linda, California; Icaria, Greece; and centenarians in the province of Ogliastra, Sardinia. These populations hold great promise for identifying key factors in the maintenance of function during aging [27,28], and several studies have highlighted isolation, lifestyle, and diet as hallmarks of longevity [29,30,31,32,33]. It is important to note that, while there are similarities between long-lived populations, there is no single formula for longevity. In addition to Sardinia, several other Italian regions have high numbers of nonagenarians and centenarians [34,35], such as Cilento, where several recent studies have focused on longevity [5,14,26,36,37]. Cilento, with a considerable number of old people and a number of factors that increase longevity, can be considered an undisclosed Blue Zone and can be compared with other Blue Zones in order to highlight factors that may be associated with increased longevity.

In this context, this narrative review aims to identify common factors between Cilento and the five LBZs, taking into account environmental, nutritional, and lifestyle factors.

## 2. Materials and Methods

**Figure 1:**
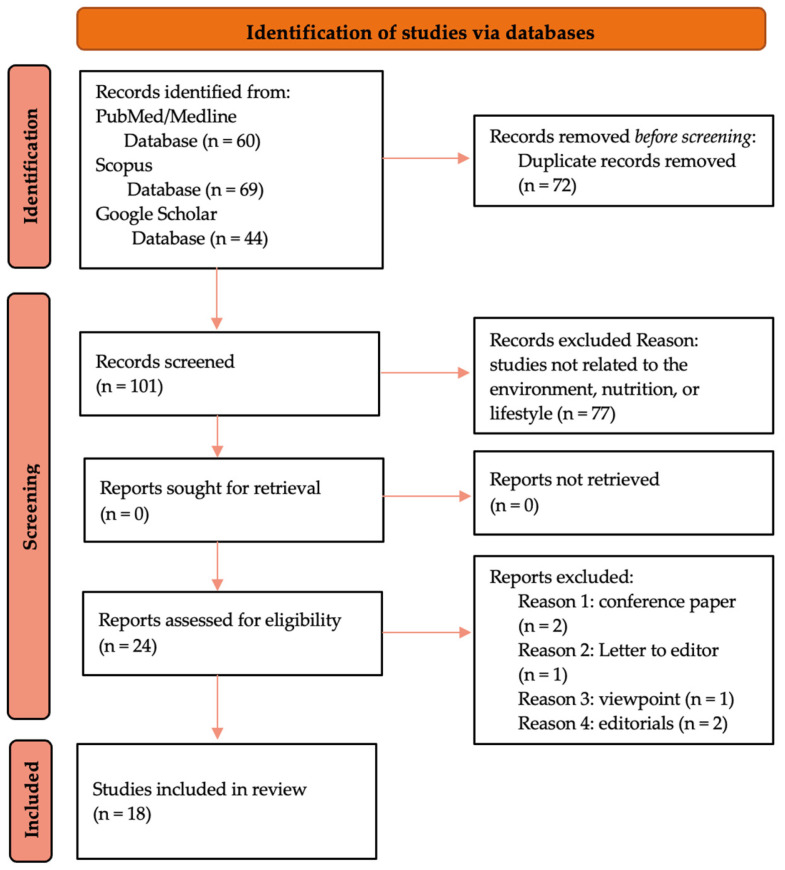
A flowchart to illustrate the selection and filtering process of articles in this narrative review (PRISMA Model Readjustment).

The literature search for potential studies in this narrative review utilized MeSH terms and keywords such as “Cilento” OR “Blue Zones” AND “longevity” AND “environmental factors” OR “lifestyle” OR “diet”. The database search strategy covered the period from 2004 to the present. This is because the term Longevity Blue Zone was first used in 2004 [38] and the international literature on this niche topic is not extensive [39]. We selected articles or chapters written in English, Italian, and German describing environmental, nutritional, and lifestyle factors in Cilento and the five LBZs from PubMed/Medline, Scopus, and Google Scholar. After removing duplicates, we reviewed the titles and abstracts of all retrieved studies. We then excluded studies that were not related to environment, nutrition, or lifestyle in Cilento and the LBZs, as well as conference papers, letters to the editor, viewpoints, and editorials. For potentially relevant studies, we obtained and reviewed the full texts before making the final selection. The co-authors discussed and agreed upon the final list of reference texts, totaling 18 (refer to Figure 1). To describe and explain the ‘similarities’ between the selected geographical areas of interest, the study utilized a descriptive comparative approach. This involved selecting items of interest, analyzing similarities, describing the selected items in their respective contexts, and, finally, possibly rectifying the categories involved in the study. 

## 3. Results

The environmental, nutritional, and lifestyle factors of Cilento and the five LBZs have been identified and explained below.

### 3.1. Cilento

Cilento is located in the Campania region of southern Italy, and part of its territory is listed as a UNESCO World Heritage Site. It covers an area of about 490,000 hectares and includes 102 municipalities. From an ecological point of view, it is a heterogeneous territory, characterized by the integration of different environments, including coastal, hilly, and mountainous areas [5], as well as the mouths of major rivers, the latter thanks to karst phenomena with favorable lithological features [40,41]. Springs are scattered in hilly and coastal areas [26,35,42]. In this area, humans have been able to integrate themselves harmoniously with the forms of the landscape [43] in a rural, agricultural, and pastoral civilization [5]. The population of Cilento is about 278,093, and the percentage of old people (40.31 per 1000) is relatively high [44]. The prevalence ratio of centenarians is 12.49 [14]. The long life expectancy in Cilento has been found to be most pronounced in the central municipalities of the area, from where it spreads to the southeastern part of the territory, according to age groups [5]. Aliberti et al. [5] reported that centenarians are found in the heart of the defined area, at an altitude between 440 and 600 m above sea level (hilly area). The region has a transitional climate between the Mediterranean and the temperate zone, with mild temperatures of about 20 degrees Celsius, rather humid winters, and moderately dry summers [5]. The mild climate seems to be an important factor in the phenomenon of longevity in Cilento. The beneficial effects of hill and mountain climates have been confirmed by Mathieu et al. [45], who mainly traced the genesis of medical interest in high-altitude climates, and by Wyder [46], who showed how mountain climates are considered therapeutic agents capable of reducing the risk of cardiovascular diseases. Some studies suggest that the appropriate temperature for the body is between 18 and 20 °C, with regions with longer life expectancy, such as Cilento [5,47,48], having an average temperature of 20 °C. Furthermore, Aliberti et al. [5] found that the old, very old, and nonagenarians of Cilento were significantly associated with UNESCO World Heritage municipalities. It is important, but little studied, how this protected natural area can influence longevity. However, the UNESCO World Heritage Site includes a Mediterranean park par excellence, where grows an olive tree from which is produced extra virgin oil (EVO) [49], with protected designation of origin (PDO), which contains substances that may play an important antioxidant role [50] and a preventive role in neoplastic and inflammatory processes, cardiovascular diseases, and metabolic disorders [51], and which may be determinants of good health and protective for the longevity groups in Cilento, in agreement with Pes et al., who showed in their study that a higher intake of olive oil has a beneficial effect on self-perceived health, physical performance, and sensory organ function [27]. In this area, protected by UNESCO, in addition to olive trees, there are also figs of the “Dottato” variety, from which the “fico bianco del Cilento” is derived. In the Cilento tradition, the white fig is dried to be eaten in winter [52]. This fruit was called “poor man’s bread” because it was the meal of the Cilento peasants who went to the fields early in the morning. Since ancient times, the fig fruit has been considered a symbol of longevity due to its antioxidant properties and potential effects on human health [53]. 

Surrounded by an ideal geomorphology and a healthy and mild climate [43], the rural area of Cilento has allowed the cultivation of Aloe Vera. According to several studies [54,55,56], Aloe Vera gel is used as a food with beneficial properties, such as anti-inflammatory, antioxidant, antiviral, and antibacterial properties, with other antihypertensive, antidiabetic, anti-obesity, and cardioprotective effects. According to our hypothesis, these local productions, listed above, in protected areas can promote longevity in the Cilento communities.

Therefore, the rural area of Cilento, where longevity is located, is “different from the other regions of Italy, both for cultural traditions and for the availability of typical products” [57] (p. 2), which allow the creation of a unique traditional cuisine. For example, Caggiano presents as a typical dish “tagliatelle con i fiori di zucca” (tagliatelle with pumpkin flowers); Sicignano degli Alburni presents “lagane con la mollica” (lagane with breadcrumbs); Controne offers “minestra di fagioli e cardoncelli” (bean and cardoncelli soup) and “carciofi farciti” (stuffed artichokes); and typical of Buccino is “gallo imbottito” (stuffed cockerel) [43]. A typical dish of the rural tradition of Cilento is the soup “strinta”. The vegetables used are usually bitter greens, depending on availability: chicory, chard, cardoon, borage, and escarole, with the addition of boiled potatoes (Table 1). These recipes are composed of macro- and micronutrients associated with the Mediterranean diet and represent a model of “healthy eating” [43,58]. Several studies have confirmed that people who follow a Mediterranean diet have a lower risk of cardiovascular disease, metabolic syndrome, obesity, and cancer; lower rates of diabetes; and less cognitive dysfunction [59,60]. It should also be remembered that in the 1960s, the American cardiovascular scientist Ancel Keys defined the concept of the Mediterranean diet in Cilento and proposed it as a key factor of healthy aging in the region [60]. 

A number of studies in the Cilento region have found that longevity is associated with the adoption of behaviors that are typical of rural areas. For example, Scelzo and colleagues [36] found that those living in the rural hills of the area adopted a way of life that included hard work, love of the land, family, and religion, which allowed them to maintain mental well-being and made them particularly resilient and optimistic. The study by Pizza et al. [26] on the nonagenarians of Cilento also emphasized the importance of lifestyle and diet, but also the importance of individual personality factors.

### 3.2. Okinawa

Okinawa Island is the southernmost prefecture of Japan and is part of the Ryukyu Archipelago. It covers an area of about 226,500 hectares. The northern part of the island is covered with forests, has a mountainous terrain, and is rich in igneous rocks. The south-eastern part of the island has only gentle limestone slopes [61]. This is where most of the population centers are located. The climate of the island is subtropical. It is hot and humid, with relatively strong seasonal tropical storms [61]. The island has two growing seasons, making it ideal for the production of fresh vegetables. Okinawans are known for their long life expectancy, high number of centenarians, and consequently low risk of age-related diseases in a population of approximately 1,285,000 [39]. Much of Okinawa’s longevity advantage is attributed to a healthy lifestyle, including the traditional diet [62,63], which is low in calories but rich in nutrients, especially vitamins, minerals, and phytonutrients in the form of antioxidants and flavonoids, many of which have nutraceutical potential. Compared to the Japanese diet, the traditional Okinawan diet shares similarities, including high vegetable and soy intake, low fat intake, preference for miso, an abundance of fish and seafood, and the absence of dairy products, but differs drastically in some key areas [62,63]. For example, the ubiquitous sweet potato, rather than rice or other grains, is the staple of the Okinawan diet. Sweet potatoes, green leafy or yellow root vegetables, and soybeans (e.g., miso soup, tofu, or other incarnations of this legume), which accompany almost every meal, are the focus of traditional Okinawan cuisine. These staples are often accompanied by small portions of fish, noodles, or lean meats seasoned with herbs, spices, and cooking oil [62,64]. Mulberry leaves cure sore throats, squid ink soup detoxifies, seaweed and ginseng are known as anti-inflammatories, goya is able to lower blood sugar levels and may explain the lower incidence of diabetes, and tofu (soybean curd) has multiple health benefits [65]. The sweet potato is the healthiest of all vegetables, primarily because it is high in fiber, naturally occurring sugars, slow-digested low-glycemic carbohydrates, protein, antioxidant vitamins A and C, potassium, iron, and calcium and is low in fat (especially saturated fat), sodium, and cholesterol. Recognizing the value of a healthy diet in reducing the risk of chronic disease, the American Cancer Society, the American Heart Association, and other organizations have also endorsed the sweet potato for its nutritional properties that may help reduce the risk of chronic age-related diseases, such as cancer and cardiovascular disease [62,66].

Okinawan-style miso soup is the start of a typical meal. Unlike the Japanese version, Okinawans prefer to garnish their miso soup with small amounts of tofu, fish, pork, or vegetables. “Champuru”, “nbushi”, and “irichi” are the three main styles of cooking. The main course is usually a “champuru” (stir-fried) vegetable dish. Vegetables such as bitter gourd, cabbage, bamboo shoots, etc., are accompanied by a garnish such as “konbu seaweed” (konbu seaweed is high in iodine, which is necessary for growth, and is an excellent source of glutamate). They are usually cooked with a little oil or pork fat, bonito broth (for flavor), and a little cooked fish or pork. In the “nbushi” style, watery vegetables, such as daikon (a type of large white radish), Chinese okra, carrots, or pumpkin, are flavored with miso and cooked in their own juices. The “Irichi” style uses a combination of boiling and frying and focuses on vegetables that are less watery. Favorite vegetables include burdock, seaweed, dried daikon, and green papaya. Freshly brewed “sanpin” (jasmine) tea is typically served, sometimes followed by locally produced awamori (millet brandy) [62,64].

The extraordinary longevity of the Okinawans has also been associated with a calorie-restricted diet [66,67]. Because the life-extending effects of caloric restriction have been observed in yeast and animals across the evolutionary spectrum [68,69,70], it is hypothesized that they are applicable to humans [39]. Suzuki [71] reported an average daily energy intake of 1407 kcal/day for men and 1096 kcal/day for women, while a study by Willcox et al. [63] reported an average daily energy intake of less than 2000 kcal/day for a 70-year-old man. In any case, it is likely that the average energy intake of Okinawans has changed over time, and it is hard to believe that the new generations born after World War II still adhere to a caloric restriction comparable to that of previous generations [39]. It should also be noted that drinking water in Okinawa is rich in minerals, especially calcium, due to the presence of limestone from coral reefs in Okinawa’s soil [72], and water rich in calcium and magnesium is associated with lower cardiovascular mortality [73].

As a way of life, before eating, people say “Hara Hachi Bu”, which means “8 out of 10”, or stop eating when the stomach feels 80 percent full. This has led to a lower incidence of obesity in Okinawa [65]. In rural areas, people live with low furniture, so they have to get up and down all the time, so they exercise by strengthening the trunk and lower body and improving balance (like doing squats). Everyone has a garden. This is a great place for daily light exercise [65]. There are groups of people called “moai” who are socially active and engage in fundraising and help each other in times of need. It is all friends getting together and talking. In America, loneliness can take 15 years off your life expectancy. In Okinawa, those years are gained simply by making friends, caring for them, and spending time with them every day. Ikigai is a mission, a purpose. It is the foundation of the well-being of the life in Okinawa. In Japan, people do not talk about retirement. They always keep their minds busy and their bodies moving. This is the meaning of their lives; it constitutes their essence. The key to longevity is in the little things [65] (Table 1).

**Table 1:** Cilento and LBZ basic characteristics.

	Cilento	Okinawa LBZ	Sardinia LBZ	Ikaria LBZ	Nicoya LBZ	Loma Linda LBZ
Population	278,093 [5]	1,285,000 [39]	1,578,146 [74]	8300 [39]	161,000 [39]	25,129 [75]
Ethnic groups	White Caucasians ***	Asian [39]	White Caucasians [39]	White Caucasians [39]	Native Americans, White Caucasians [39]	White non-Hispanic, Asian, White Hispanic ***
Altitude *	400–600 m [5]	503 m ***	554 m [76]	457 m ***	400 m [77]	355 m ***
Climate	Mediterranean 20 °C mean [5]	Subtropical 23.55 °C [28]	Mediterranean 23.5 °C [28]	Mediterranean 19.3 °C [78]	Tropical 25 °C [79]	Mediterranean 17.4 °C [80]
Traditional occupation	Agriculture, animal husbandry [14]	Agriculture [39]	Agriculture, animal husbandry [39]	Agriculture, animal husbandry [39]	Agriculture, forestry work [39]	Volunteering [81]
Religion	Catholic ***	Ryukyuana (ancestral cult, adoration) [81]	Catholic ***	Orthodox Christian ***	Catholics 85% Atheists 11% Others 4% ***	Adventist [82]
Family	Strong relations [24,36]	Relationships between generations [65]	Relationships between generations [65]	Relationships between generations [83]	Relationships between generations [81]	Relations [84]
Social activities	Community relationships [24,36]	Community relationships [81]	Community relationships [65]	Community relationships [83]	Community relationships [81]	Community relationships [84]
Daily energy intake per capita (kcal)	2250 [57]	<2000 [63]	2600 [39]	<1500 [39]	2392 [39]	n
Nutrition **	Mediterranean diet [60]	Plant-based [65]	Mediterranean diet [65]	Mediterranean diet [65]	Traditional Mesoamerican [65]	Whole foods, plant-based [65]
Typical products and meals	“Dottato” white figs [52], extra virgin olive oil, red wine, honey [5,43] “**Strinta**” soup, made with chicory, chard, cardoons, borage and escarole, boiled potatoes, EVO oil, garlic clove and chili pepper, beans can be added [43]	Tofu, vegetables, purple potatoes [81] “**Champuru**”, made with bitter gourd, cabbage, bamboo shoots, accompanied by “konbu seaweed “**Nbushi**”, watery vegetables such as daikon, Chinese okra, carrots, or squash, seasoned with miso and cooked in their own juices “**Irichi**”, a combination of slow-cooked and stir-fried vegetables, including burdock, seaweed, dried daikon, and green papaya [64]	Pasta, sourdough bread, vegetables, beans and whole grains, red wine [81] Traditional “**minestrone**”, made with onions, fennel, carrots, legumes (beans, broad beans, peas), potatoes, and bacon [81]	Olive oil, vegetables, fruit, legumes, local products, red wine, herbal tea, honey [81] “**Soufiko**”, made with eggplant, yellow pepper, potatoes, green beans, tomatoes, onions, garlic, zucchini, chopped red pepper, extra virgin olive oil, red wine ***	Beans, squash, and corn, called “the three sisters”; rosquillas; tortillas; fruits such as mango and papaya [81] “**Gallo pinto**”, a mixture of rice and black beans, often accompanied by corn tortillas ***	Fruits; vegetables; very little red meat, chicken, and fish [81]

* Altitude where most of the centenarians are concentrated. ** How centenarians ate for most of their lives. *** The authors developed this information from various sources. Bold is used to distinguish recipe names from components.

### 3.3. Sardinia

Sardinia is an island in the south of Italy, surrounded by the Mediterranean Sea. An ecologically heterogeneous area characterized by the integration of different environments, including coastal, hilly, and mountainous areas, and by the presence of lakes, with a total surface area of 2,410,000 hectares. The area of greatest longevity, identified in the hilly part of Sardinia, comprises the group of six municipalities in the provinces of Barbagia and Ogliastra, with a total surface area of 315,500 ha, and the municipality of Villagrande-Strisaili, which represents the center of the LBZ [85]. Sardinia has a population of about 1578,146 [74] people, and the prevalence ratio of old people is 5.91. The prevalence ratio of centenarians is 0.37. They are mainly engaged in pastoral and agricultural activities and live a relatively traditional lifestyle. These populations have been isolated for centuries, which has contributed to making their genetic heritage more homogeneous and preserving their sociocultural and anthropological characteristics throughout their history [86]. Non-genetic factors, such as physical activity [87,88], life satisfaction, optimism, resilience, religiosity [88], and diet, may explain the exceptional life expectancy recorded in central Sardinia [27,76]. The modern Sardinian diet has its historical origins in a mixture of agricultural and pastoral traditions [89] and can be considered a variant of the Mediterranean diet, with a slightly higher consumption of animal products, such as cheese, meat, and lard.

The main staple of the diet is whole-grain bread [27,89]. The process of making bread (simple carbohydrates) is very different from ordinary white bread because it is leavened with a bacterium called Lactobacillus, which is capable of reducing the glycemic load of an entire meal. Maioli et al. [90] showed that sourdough bread was able to reduce postprandial glucose levels by 25%. In addition, many complex carbohydrates, such as whole grains, vegetables, and beans, are consumed. The traditional minestrone, made with onions, fennel, carrots, legumes (beans, broad beans, and peas), and lots of potatoes, is also very popular. To the traditional soup, however, are added pieces of fried bacon. In Sardinia, carbohydrate consumption has been associated with improved life satisfaction [91] and reduced diabetes. Passeri et al. [92], who studied bone metabolism in 104 subjects aged 98 to 105 years, found that 99 of the 104 subjects were vitamin D deficient and that this was the cause of the high incidence of fragility fractures in centenarians. In contrast, the Sardinian diet (rich in whole grains, beans, and vegetables) contains high levels of vitamin D, which is essential for the skeletal, immune, and other systems [93], as well as choline, which prevents age-related memory decline and protects the brain from neuropathological changes associated with Alzheimer’s disease [94]. 

Corder et al. [95] suggested that red wine consumption may be associated with reduced cardiovascular mortality in Sardinians due to its high resveratrol and proanthocyanidin content. Meanwhile, Biasi et al. [96] demonstrated in an in vitro model of human Caco-2 enterocytes that phenolic compounds present in local red wine extracts can counteract age-related inflammation by reducing proinflammatory cytokine release. In Sardinia, red wine is consumed regularly and in moderation with meals, as is typical of the Mediterranean diet [97,98].

The family plays a fundamental role for older people In Sardinia. Nursing homes are not considered, and older people are usually cared for by their daughters [65] (Table 1).

### 3.4. Ikaria

Located in the Eastern Aegean, between Samos and Mykonos, the island of Ikaria is part of the Eastern Sporades. It covers an area of 25,532 hectares and is divided into three municipalities: the municipality of Saint Kirykos, which is the capital and the southern port of the island; the municipality of Evdilos, which is the northern port; and the municipality of Rahes, which is located in the central–western part of the island. Ikaria is entirely composed of crystalline schist of metamorphic origin. The topography varies from green slopes to barren and rugged cliffs. Most of the island is mountainous. Due to the geological and tectonic structure, the island has rich aquifers and many springs [99,100]. Most of the villages are located in the lowlands close to the coast at an altitude of about 457 m above sea level, while some are located in the mountains [101,102]. The island’s climate is typically Mediterranean. The average annual temperature is 19.3 °C [78]. Most of Ikaria is included in the NATURA 2000 network for nature conservation due to its biophysical diversity [98]. With a population of approximately 8300, Ikaria has a life expectancy comparable to other long-lived populations [28]. Historical documents, such as the 17th century reports of Archbishop Joseph Georgirenes, noted the exceptional health and longevity of the islanders, attributing it to the quality of the air and water [103].

The Ikarians had to learn how to survive in a barren, rocky place, and this stimulated a resilience not seen in other places. They had to develop the ability to live off the land; to recognize plants as food sources, as vegetables, as herbs, and even as medicines; to harness bees; and, above all, to cooperate in the hardest of times. The population, though small, has maintained relatively good health despite factors such as low socioeconomic status and the presence of cardiovascular risk factors [99].

Agriculture is the most common occupation in Ikaria, which is not surprising given the island’s location, and goat meat, milk, and cheese are staples [100]. The diet is typically Mediterranean, rich in olive oil, vegetables, fruits, legumes, and local products (Figure 1). Locally produced olive oil, with its higher concentration of polyphenols, has been associated with vascular protection [104]. 

Over time, Ikarians developed the habit of drinking infusions of local herbs, especially sage tea, rosemary tea, and mallow tea. Drinking herbal teas has been associated with a number of health benefits, including a reduction in the incidence of dementia. Herbal teas also have anti-inflammatory and antioxidant properties, and they often contain diuretics that lower blood pressure. The extraordinary longevity of the island is linked to the habit of drinking herbal teas. Honey has been used in Ikaria for thousands of years. Beehives are set up among the pine forests on the higher altitudes. Honey is rich in micronutrients and bioactive compounds, as there is no pasteurization of Ikarian honey, and scientific studies show that honey has anticancer properties [65]. Historically, wine played a surprising role in Ikarian longevity culture. On the other hand, Greek mythology says that wine was born in Ikaria. The same grapes and even the same ancient process have been used for centuries. The wine of Ikaria is different; it is a natural wine without any added chemicals. Potassium, phosphorus, boron, and iron are the minerals it contains. These minerals, combined with the microclimate and the quality of the grapes, give the wine unique qualities, so much so that it is defined as a medicinal wine. The ability to assimilate antioxidants has been proven to be increased by the combination of a Mediterranean diet and Ikarian wine. In Ikaria, the same wine has been the drink of the people for more than 100 generations, and the people have a very long-life span [65].

Although the homes are isolated, Ikarians have high levels of family solidarity, interaction, and frequent contact with neighbors, family, and friends [83]. In long-term relationships, when one spouse dies, the other spouse is about two-thirds more likely to die within the next three months. Residents of Blue Zones prioritize their companions and nurture their relationships [65]. Religious participation, especially during significant events, is widespread. It is positively associated with mental and physical health [105].

Physical activity is a fundamental aspect of the Ikarian lifestyle, taking the form of farming and walking in mountainous terrain. Adherence to the Mediterranean diet, combined with regular physical activity, contributes to good health [79] (Table 1).

### 3.5. Nicoya

Located in the northeastern part of Costa Rica, on the Pacific Ocean, is the Nicoya Peninsula. Nicoya covers an area of 31,060 hectares in the province of Guanacaste. The landscape of the region is characterized by tropical dry forests and pastures and a tropical climate with a dry season from December to April and a rainy season from May to November [106]. The peninsula includes the following five neighboring cantons: Santa Cruz, Carrillo, Nicoya, Nandayure, and Hojancha, with altitudes ranging from 100 to 500 m. Nicoya has an altitude of 400 m above sea level. The total population of the five cantons is 161,000, of which 47% live in urban areas, mostly in three small towns of about 25,000 people—Nicoya, Santa Cruz, and Philadelphia—according to the 2011 census. Eight percent of the population is 65 years of age or older. According to the 2011 census, Nicoya had 32 centenarians, and the overall mortality rate was 20% lower than in the rest of the country [77,107]. The longevity advantage is more pronounced among men and is attributed to a lower incidence of cardiovascular disease [108]. A study of Nicoya’s older population found a significantly lower mortality ratio of 0.71 compared to the rest of Costa Rica, indicating exceptional life expectancy [109]. Although the indigenous population in Costa Rica is quantitatively small (2%, according to the 2011 census), the indigenous population in Nicoya is larger: 5% reported belonging to the Chorotega ethnic group. Stanford scientists, Rehkopf et al. [108], calculated the biological age of Nicoya’s inhabitants based on telomeres and found that Nicoya’s inhabitants have a biological age that is about ten years younger than their chronological age [108].

What explains this gap in Nicoya? Costa Ricans have an expression that indicates their purpose, “plan de vida”; they have a sense of direction, they have a purpose in life, and this is what drives them to overcome difficulties [81].

**Figure 2:**
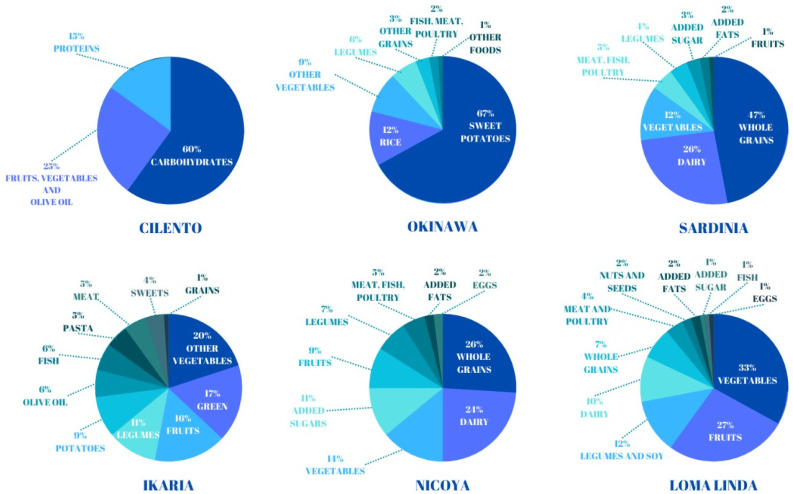
Dietary variations in Cilento (reproduction from data on the Mediterranean diet) and the LBZs (reproduction from data by Dan Buettner).

Rosquillas are a typical dish in Nicoya. One of the reasons for the long life in Nicoya is the diet of beans, squash, and corn, called “the three sisters”. It is the triad of the Mesoamerican diet that has been consumed by the inhabitants of this region for at least 6000 years. Corn, often in the form of tortillas, is one of the staples of traditional cuisine (Table 1). The kernels are an excellent source of complex carbohydrates, rich in vitamins, minerals, and fiber. And the classic preparation of corn increases its nutritional value, starting with the ash that women add when they soak the corn. The ash breaks down the cell walls of the kernels, releasing niacin, which helps control cholesterol. Black beans contain the same antioxidants as blueberries and are rich in fiber, which helps cleanse the colon. Pumpkin is a good source of vitamins A, B, and C and is rich in minerals such as potassium and magnesium [81]. Like other tropical countries, the Nicoya diet is rich in fruits, such as mango and papaya. The study by Momi-Chacon and others [110] showed that 74% of Nicoyans between the ages of 90 and 109 eat fruit one to three times a day and the rest two to six times a week. In addition, 88% of participants consumed tubers (potatoes, sweet potatoes, and cassava) two to three times per week (Figure 2). However, the carbohydrate foods consumed by the Nicoyans had a low glycemic index, which may have delayed disease onset [107]. Nicoya’s calcium- and magnesium-rich drinking water may have reduced the population’s cardiovascular mortality rate [111,112].

Agriculture and pastoralism are the most common occupations in Nicoya, with a predominantly plant-based diet and low consumption of red meat [81]. The indigenous population of the region, especially the Chorotega ethnic group, which makes up 5 percent of the population, influences the local lifestyle and behavior [113]. The lifestyle is characterized by traditional occupations; Nicoyans do everything by hand; there are no machines for housework or gardening. They use machetes to cut grass and split wood, grind corn by hand, and carry out household activities, gathering and preparing food, and this involves unconscious movements that end up being more physical activity than exercise. They are very active in the morning and rest in the afternoon [81].

Religiously and socially, the people of Nicoya have a strong sense of community and are deeply rooted in their traditions. The social fabric of the region is very dense, fostering strong interpersonal relationships and community ties that contribute to the overall well-being and longevity of its inhabitants [81].

### 3.6. Loma Linda

Loma Linda is not an exotic place, but a small town in the middle of an American province, located about a hundred kilometers from Los Angeles, between the peaks of San Bernardino and San Jacinto. Loma Linda is in a strategic position between beaches, mountains, and desert. It covers an area of 195,500 hectares and has an altitude of 355 m above sea level and a Mediterranean climate with an average annual temperature of 17.4 °C [39,65,80,81]. The population is about 25,129 inhabitants [75]. It is predominantly a Seventh-Day Adventist community [82]. Charlemagne-Badal and Lee [114] found that, among those who survived past the age of 35, Adventist women in California lived 3.7 years longer than their counterparts and Adventist men lived 6.2 years longer. The differences were even more pronounced—4.4 years for women and 7.3 years for men—in a later, larger sample from Fraser and Shavlik’s research [115].

Loma Linda Adventists have found ways to maintain a consistent program of physical activity and other healthy habits [81]. Exercise, a vegetarian diet, not smoking, eating nuts, and having social support have been shown to predict longevity among Adventists [116].

They are united in this doctrine of wellness, where the difference is not so much the physical environment as the religious and social environment. Adventists adhere to a set of behavioral pillars that they believe will bring them closer to God. They are associated with religious devotion [81]. Several studies have highlighted the relationship between physical and mental health and religion or spirituality as a positive factor [117,118,119,120]. In fact, Hall [121] concluded that religious attendance is more cost-effective in terms of increased longevity than statin-type medications. In line with this, Hummer and his colleagues [122] concluded that there is consistent evidence that attendance at religious services is associated with a lower risk of death.

Another mechanism by which religion may be associated with physical health is through social support. Social integration is facilitated by religious organizations: religious groups also often have formal assistance programs for those with problems with finances, activities of daily living, or major life transitions [84]. Finally, religious communities regularly bring together people with shared values, commitments, and goals. This helps create a sense of connectedness and support [84].

Another element common to all Adventists is the importance they place on nutrition. A healthy lifestyle is not only for health, but also to maintain a clear mind and improve one’s relationship with the Divine. The founder of Adventism instituted the elimination of meat from the diet and established a balanced vegetarian diet that included fruits, legumes, grains, vegetables, and dried fruits. With a vegetarian diet, resistance increases and cholesterol decreases [81] (Table 1).

Statistics show that 35% of Adventists are vegan or vegetarian. Adventists eat a lot of fruits and vegetables, which constitute 60% of their diet, and only 5% of calories come from red meat, poultry, and fish. The Adventist study clearly points the way to a vegetarian diet [81] (Figure 2).

## 4. Discussion

Taking into account environmental, nutritional, and lifestyle factors, this narrative review aimed to identify the similarities between Cilento and the five LBZs. The similarities and differences are presented below to determine if Cilento could be considered an undefined LBZ.

In Italy, Cilento has characteristics in common with the LBZs, and although these places seem to be very different geographically, some being islands, some hilly, some very remote, while others are surprisingly urban, they all have the same common denominators. They all have, more or less, the same environment, the same diet, and the same lifestyle formula that can produce the longest-living people on the planet.

First of all, from an environmental point of view, Cilento and the LBZs have the same hilly altitudes [cf. 5], ranging from a minimum of 355 to a maximum of 600 m above sea level. This finding is in agreement with the results of our previous study [5], where long-lived people in Cilento were found to be concentrated at altitudes between 400 and 600 m asl. Moreover, these areas are mostly characterized by a Mediterranean climate (Cilento, Sardinia, Ikaria, and Loma Linda) with mild average annual temperatures ranging from 17.4 °C to 23.5 °C; these temperatures also characterize regions with tropical (Nicoya) and subtropical (Okinawa) climates. A mild climate seems to be an important factor for the longevity phenomenon in Cilento and the Blue Zones. Several studies confirm that the right temperature for the body is between 18 °C and 20 °C, and that regions with a longer-lived population, such as Cilento, have an average temperature around 20 °C [5,47,48]. In particular, the beneficial effects of hill and mountain climates have been confirmed by Mathieu et al. [45], who mainly traced the genesis of medical interest in high-altitude climates. In addition, a mild climate allows the cultivation of many vegetables, fruits, and legumes (in Cilento and the Blue Zones) and the production of olive oil and wine production, especially in Cilento, Sardinia, and Ikaria [43,81].

The diets of Cilento and the LBZs are very different due to the different historical, cultural, and culinary traditions of the populations [39,43,91]. However, they all have in common the widespread consumption of native foods, mainly of plant origin, with an abundance of fruits and vegetables, legumes, and cereals and a moderate consumption of proteins. The consumption of potatoes is characteristic of Okinawa, Sardinia, and Cilento. Although, in the literature, a diet rich in potatoes is considered less healthy than most plant-based diets [123], especially because of cooking methods used (e.g., frying), on the contrary, a recent paper highlights that, in Sardinia, potatoes are consumed boiled and seasoned with fats capable of lowering the glycemic index [27]. The same is true in Cilento, where potatoes are boiled and seasoned with extra virgin olive oil. In Okinawa, the sweet potato is one of the healthiest vegetables, mainly due to its fiber content, natural sugars, and carbohydrates with a low glycemic index [81]. It is well known that the Mediterranean diet promotes health [40], but it is also recognized that not all Mediterranean populations are equally long-lived; these data confirm that longevity is the sum of genetic and macro- and microenvironmental factors and not just the result of a single factor, such as diet. Further confirmation is provided by the significant variations in the Mediterranean model. For example, among the Mediterranean regions, in Ikaria, fewer carbohydrates and more legumes are consumed, along with vegetables and fruits, meat, fish, and poultry, while in Sardinia, more whole grains and dairy products; fewer vegetables, fruits, and legumes; and less meat are consumed than in Ikaria. In Cilento, on the other hand, more carbohydrates, fewer fruits and vegetables, and less protein are consumed [43,57]. Unique products of the three Mediterranean regions are the extra virgin olive oil with a higher concentration of polyphenols, the moderate daily use of wine with its antioxidant content, and (in Cilento and Ikaria) the use of honey, rich in micronutrients and bioactive compounds [43,81]. The culture of eating pork is shared by the LBZs of Okinawa, Sardinia, and Nicoya, although with differences in the preparation and frequency of eating the meat, while in the LBZ of Ikaria, the consumption of goat meat prevails. Sheep, goat, and, in the winter months, pork are the most common meats in Cilento. Meat consumption, both in Cilento and in the five LBZs, has always been moderate, and the animals are mostly reared in the wild, in areas with a high biodiversity, which makes the meat more nutritious and less harmful than that coming from intensive large-scale farming [39,81,124].

From a lifestyle perspective, studies conducted in Cilento and the LBZs have shown that longevity is associated with behaviors adopted in rural areas, except for Loma Linda. In fact, the older people and centenarians living in the hills have adopted a lifestyle that includes hard work, love of the land and the family, religious devotion, social integration, and a personality that allows them to maintain mental health, making them particularly resilient and optimistic [33,34,39,81,125].

**Figure 3:**
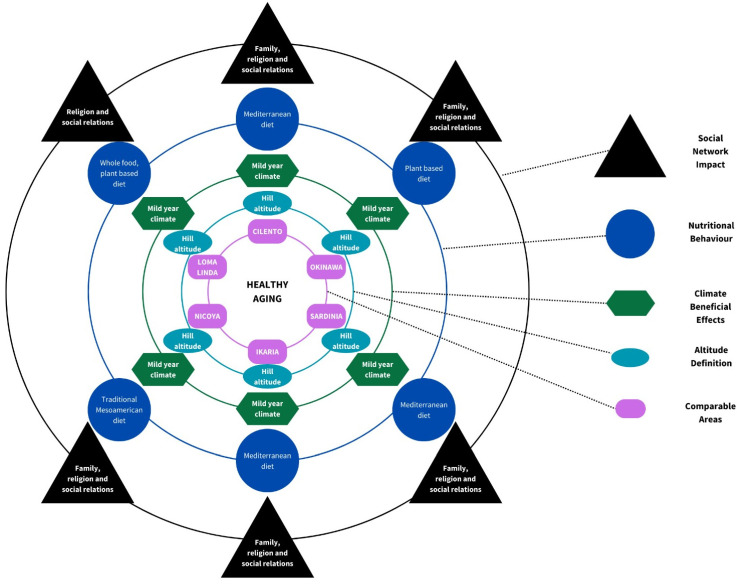
Circle model to explain the common factors between Cilento and LBZs, considering Dahlgren and Whitehead’s health determinants scheme [130].

At Loma Linda, however, they are united in the doctrine of well-being, according to which longevity is conferred not by the macroenvironment but by the religious and social environment (Figure 3).

It is not possible to quantify leisure time in Cilento and other LBZs, but what has been emphasized is that people prefer to enjoy family, friends, and rest rather than work a few extra hours. In other words, they slow down and make time for the things that really matter and are important to them.

On the other hand, people in these places live to be 100 years old with extraordinary frequency. People live vibrantly, actively, happily, and perhaps most importantly, they live longer without forcing themselves.

Future research directions in Cilento regarding environmental and nutritional factors will follow the guidelines of the UN Sustainable Development Goals (SDGs) 2030 [126] and the Strategic Plan for Biodiversity [127]. The aim will be to enhance biodiversity and agrobiodiversity in order to achieve sustainable development and possibly protect the older population from diseases. In agreement with Fara [128], who emphasized the relationship between sustainability and nutrition quality, it is important to consider healthy eating habits from different cultures; for example, Asian cuisines have an ancient tradition of maintaining health, and the Mediterranean diet has been recognized by UNESCO as an intangible cultural heritage of humanity. Last but not least, in order to assess the frequency with which the older population of Cilento consumes food and to evaluate its quality, a new Healthy Diet Index will be used, according to Ma et al. [129].

## 5. Conclusions

Longevity is not universal, and only a few regions exhibit high longevity. Therefore, it is essential to identify other areas with similar characteristics to LBZs to better understand the factors that contribute to a long and healthy life.

Cilento and the LBZs share several common factors, including a hilly altitude (ranging from 355 to 600 m), a mild climate throughout the year (with temperatures between 17.4 and 23.5 degrees Celsius), traditional professions such as agriculture and animal husbandry, a predominantly Mediterranean or plant-based diet, and typical recipes based on legumes, tubers, vegetables, and extra virgin olive oil. Additionally, strong intergenerational family relationships, religious devotion, and the desire to maintain social relationships within the community are also prevalent. Given the similarities to Cilento, one may wonder if this is an LBZ waiting to be discovered. Lessons learned from this discovery could be applied to the general population to protect them from chronic non-communicable diseases and help slow the aging process.

## Data Availability

PubMed/Medline, Scopus, and Google Scholar.

## References

[1] Willcox D.C., Willcox B.J., He Q., Wang N.C., Suzuki M. (2008). They really are that old: A validation study of centenarian prevalence in Okinawa. J. Gerontol. A Biol. Sci. Med. Sci..

[2] Kannisto V. (1994). Development of Oldest-Old Mortality, 1950–1990: Evidence from 28 Developed Countries.

[3] Jeune B., Vaupel J.W. (1995). Exceptional Longevity: From Prehistory to the Present.

[4] WPP, World Population Prospects, United Nations, Department of Economic and Social Affairs (2022). Population Division. https://population.un.org/wpp/Download/Probabilistic/Population/.

[5] Aliberti S.M., De Caro F., Funk R.H.W., Schiavo L., Gonnella J., Boccia G., Capunzo M. (2022). Extreme Longevity: Analysis of the Direct or Indirect Influence of Environmental Factors on Old, Nonagenarians, and Centenarians in Cilento, Italy. Int. J. Environ. Res. Public Health.

[6] Smith D.W. (1997). Centenarians: Human longevity outliers. Gerontologist.

[7] Puca A.A. (2004). A caccia dei geni della longevità. Le Sci..

[8] Bernstein A.M., Willcox B.J., Tamaki H., Kunishima N., Suzuki M., Willcox D.C., Yoo J.-S.K., Perls T.T. (2004). First autopsy study of an Okinawan centenarian: Absence of many age-related diseases. J. Gerontol. A Biol. Sci. Med. Sci..

[9] Fries J.F. (1980). Aging, natural death, and the compression of morbidity. N. Engl. J. Med..

[10] Evert J., Lawler E., Bogan H., Perls T. (2003). Morbidity profiles of centenarians: Survivors, delayers, and escapers. J. Gerontol. A Biol. Sci. Med. Sci..

[11] Barbi E., Caselli G., Vallin J. (2003). Hétérogénéité des générations et âge extrême de le vie. Population.

[12] Franceschi C., Passarino G., Mari D., Monti D. (2017). Centenarians as a 21st century healthy aging model: A legacy of humanity and the need for a world-wide consortium (WWC100+). Mech. Ageing Dev..

[13] Franceschi C., Motta L., Motta M., Malaguarnera M., Capri M., Vasto S., Candore G., Caruso C., IMUSCE (2008). The extreme longevity: The state of the art in Italy. Exp. Gerontol..

[14] Aliberti S.M., Funk R.H.W., Ciaglia E., Gonnella J., Giudice A., Vecchione C., Puca A.A., Capunzo M. (2023). Old, Nonagenarians, and Centenarians in Cilento, Italy and the Association of Lifespan with the Level of Some Physicochemical Elements in Tap Drinking Water. Nutrients.

[15] Eggert S., Kuhlmey A., Suhr R., Dräger D. (2018). Hundertjährige in Vorbereitung auf das Lebensende?. Z. Gerontol. Geriat..

[16] Murotani K., Zhou B., Kaneda H., Nakatani E., Kojima S., Nagai Y., Fukushima M. (2015). Survival of centenarians in Japan. J. Biosoc. Sci..

[17] Kane A.E., Sinclair D.A. (2019). Epigenetic changes during aging and their reprogramming potential. Crit. Rev. Biochem. Mol. Biol..

[18] Benayoun B.A., Pollina E.A., Brunet A. (2015). Epigenetic regulation of ageing: Linking environmental inputs to genomic stability. Nat. Rev. Mol. Cell Biol..

[19] Brian J.M., Willcox B.J., Donlon T.A. (2019). Genetic and epigenetic regulation of human aging and longevity. Biochim. Biophys. Acta Mol. Basis Dis..

[20] Spinetti G., Sangalli E., Specchia C., Villa F., Spinelli C., Pipolo R., Carrizzo A., Greco S., Voellenkle C., Vecchione C. (2017). The expression of the BPIFB4 and CXCR4 associates with sustained health in long-living individuals from Cilento-Italy. Aging.

[21] Feng Z., Lin M., Wu R. (2011). The regulation of aging and longevity: A new and complex role of p53. Genes Cancer.

[22] Dato S., Crocco P., D’Aquila P., de Rango F., Bellizzi D., Rose G., Passrino G. (2013). Exploring the role of genetic variability and lifestyle on oxidative stress response for healthy aging and longevity. Int. J. Mol. Sci..

[23] Azin A.L., Zeldi I.P., Smirnov A.V., Shagibalov R.Z. (2001). Aging and longevity as indicators of ecological health of the environment. Russ. J. Ecol..

[24] Aliberti S.M., Funk R.H.W., Schiavo L., Giudice A., Ciaglia E., Puca A.A., Gonnella J., Capunzo M. (2022). Clinical Status, Nutritional Behavior, and Lifestyle, and Determinants of Community Well-Being of Patients from the Perspective of Physicians: A Cross-Sectional Study of Young Older Adults, Nonagenarians, and Centenarians in Salerno and Province, Italy. Nutrients.

[25] Darviri C., Demakakos P., Tigani X., Charizani F., Tsiou C. (2009). Psychosocial dimensions of exceptional longevity: A qualitative exploration of centenarians’ experiences, personality, and Life strategies. Int. J. Aging Hum. Dev..

[26] Pizza V., Antonini P., Marino R., D’Arena G., Lucibello S.G., Rizzo M., Brenner D.A., Jeste D.V., Di Somma S. (2020). Cognitive Health of Nonagenarians in Southern Italy: A Descriptive Analysis from a Cross-Sectional, Home-Based Pilot Study of Exceptional Longevity. Medicina.

[27] Pes G.M., Poulain M., Errigo A., Dore M.P. (2021). Evolution of the Dietary Patterns across Nutrition Transition in the Sardinian Longevity Blue Zone and Association with Health Indicators in the Oldest Old. Nutrients.

[28] Poulain M., Herm A., Pes G.M. (2013). The blue zones: Areas of exceptional longevity around the world. Vienna Yearb. Popul. Res..

[29] Robine J.M., Herrmann F.R., Arai Y., Craig Willcox D., Gondo Y., Hirose N., Suzuki M., Saito Y. (2013). Accuracy of the centenarian numbers in Okinawa and the role of the Okinawan diet on longevity: Responses to Le Bourg about the article “Exploring the impact of climate on human longevity”. Exp. Gerontol..

[30] Poulain M., Herm A., Errigo A., Chrysohoou C., Legrand R., Passarino G., Stazi M.A., Voutekatis K.G., Gonos E.S., Franceschi C. (2021). Specific features of the oldest old from the Longevity Blue Zones in Ikaria and Sardinia. Mech. Ageing Dev..

[31] Liu T., Gatto N.M., Chen Z., Qiu H., Lee G., Duerksen-Hughes P., Fraser G., Wang C. (2020). Vegetarian diets, circulating miRNA expression and healthspan in subjects living in the Blue Zone. Precis. Clin. Med..

[32] Legrand R., Nuemi G., Poulain M., Manckoundia P. (2021). Description of Lifestyle, Including Social Life, Diet and Physical Activity, of People ≥ 90 years Living in Ikaria, a Longevity Blue Zone. Int. J. Environ. Res. Public Health.

[33] Matsukasi T., Hishinuma S. (1988). Examination of centenarians and factors affecting longevity in Japan, chapter. Why do the Japanese Live Long?.

[34] Vasto S., Buscemi S., Barera A., Di Carlo M., Accardi G., Caruso C. (2014). Mediterranean Diet and Healthy Ageing: A Sicilian Perspective. Gerontology.

[35] Roli G., Samoggia A., Miglio R., Rettaroli R. (2012). Longevity pattern in the Italian region of Emilia-Romagna: A dynamic perspective. Geospat. Health.

[36] Scelzo A., Di Somma S., Antonini P., Montross L.P., Schork N., Brenner D., Jeste D.V. (2017). Mixed-methods quantitative–qualitative study of 29 nonagenarians and centenarians in rural Southern Italy: Focus on positive psychological traits. Int. Psychogeriatr..

[37] Daniels L.B., Antonini P., Marino R., Rizzo M., Navarin S., Lucibello S.G., Maisel A.S., Pizza V., Brenner D.A., Jeste D.V. (2020). Cardiovascular health of nonagenarians in southern Italy: A cross-sectional, home-based pilot study of longevity. J. Cardiovasc. Med..

[38] Poulain M., Pes G.M., Grasland C., Carru C., Ferrucci L., Baggio G., Franceschi C., Deiana L. (2004). Identification of a geographic area characterized by extreme longevity in the Sardinia Island: The AKEA study. Exp. Gerontol..

[39] Pes G.M., Dore M.P., Tsofliou F., Poulain M. (2022). Diet and longevity in the Blue Zones: A set-and-forget issue?. Maturitas.

[40] Ente Parco Nazionale del Cilento, Vallo di Diano e Degli Alburni Geomorfologia e Geologia. http://www.cilentoediano.it/it/geomofologia-geologia.

[41] Li X., Liu Z., Yao Y., Liu Y.-M., Guo D.-M., Ju W., Wu G.-R., Li Z., Guo X.-B. (2016). Comparison of the mineral elements in drinking water between Mengshan longevity district and Jinan city. Trace Elem. Electrolytes.

[42] Gambino R., Nicoletti D., Rossi F., Blasi C., Milone M., Pasca R., Quaranta G., Cillo B., Coppola P., Amendol A. Parco Nazionale del Cilento e Vallo di Diano. Relazione illustrativa. Regione Campania, Giunta Regionale–delibera n. 617 del 13 aprile 2007. https://www.yumpu.com/it/document/view/16186693/piano-del-parco-relazione-illustrativa-parksit.

[43] Aliberti S.M., Ammaturo N. (2012). Produzioni locali e tradizione gastronomica: Recupero e valorizzazione. Tra Vulnerabilità e Resilienza. Immagini di Transizione Socio-Ecologica in Un’area Della Campania.

[44] ISTAT Popolazione Residente 2020. Regioni e Comuni; Istituto Nazionale di Statistica: Roma, Italy, 2021. https://dati.istat.it/Index.aspx?DataSetCode=DCIS_POPRES1.

[45] Mathieu J., Boscani Leoni S. (2005). Die Alpen! Zur Europäischen Wahrnehmungsgeschichte Seit der Renaissance.

[46] Wyder M. (2003). Kräuter, Kröpfe, Höhenkuren: Die Alpen in der Medizin, die Medizin in den Alpen.

[47] Cheng Q.D. (1998). The climate has an effect on health. Lib. Army Health.

[48] Lv J., Wang W., Li Y. (2011). Effects of environmental factors on the longevous people in China. Arch. Gerontol. Geriatr..

[49] Assessorato Agricoltura Prodotti Titpici Della Campania. Cilento (Olio Extravergine di Oliva) D.O.P. http://www.agricoltura.regione.campania.it/tipici/olio-cilento.html.

[50] Corominas-Faja B., Santangelo E., Cuyàs E., Micol V., Joven J., Ariza X., Segura-Carretero A., Garcìa J. (2014). Computer-aided discovery of biological activity spectra for anti-aging and anti-cancer olive oil oleuropeins. Aging.

[51] De Santis S., Cariello M., Piccinin E., Sabbà C., Moschetta A. (2019). Extra Virgin Olive Oil: Lesson from Nutrigenomics. Nutrients.

[52] Russo F., Caporaso N., Paduano A., Sacchi R. (2014). Phenolic compounds in fresh and dried figs from Cilento (Italy), by considering Breba crop and full crop, in comparison to Turkish and Greek dried figs. J. Food Sci..

[53] Avarniti O.S., Samaras Y., Gatidou G., Thomaidis N.S., Stasinakis A.S. (2019). Review on fresh and dried figs: Chemical analysis and occurrence of phytochemical compounds, antioxidant capacity and health effects. Food Res. Int..

[54] Shakib Z., Shahraki N., Razavi B.M., Hosseinzadeh H. (2019). *Aloe vera* as an herbal medicine in the treatment of metabolic syndrome: A review. Phytother. Res..

[55] Kar S.K., Bera T.K. (2018). Phytochemical constituents of aloe vera and their multifunctional properties: A comprehensive review. Int. J. Pharm. Sci. Res..

[56] Ferro V.A., Bradbury F., Cameron P., Shakir E., Rahman S.R., Stimson W.H. (2003). In Vitro susceptibilities of *Shigella flexneri* and Streptococcus pyogenes to inner gel of *Aloe barbadensis* Miller. Antimicrob. Agents Chemother..

[57] Ferro M., Lucarelli G., Buonerba C., Terracciano D., Boccia G., Cerullo G., Cosimato V. (2021). Narrative review of Mediterranean diet in Cilento: Longevity and potential prevention for prostate cancer. Ther. Adv. Urol..

[58] Tyrovolas S., Polychronopoulos E. (2011). Lessons from Studies in Middle-Aged and Older Adults Living in Mediterranean Islands: The Role of Dietary Habits and Nutrition Services. Cardiol. Res. Pract..

[59] Guasch-Ferré M., Willett W.C. (2021). The Mediterranean diet and health: A comprehensive overview. J. Intern. Med..

[60] Melander O., Antonini P., Ottosson F., Brunkwall L., Gallo W., Nilsson P.M., Orho-Melander M., Pacente G., D’Arena G., Di Somma S. (2021). Comparison of cardiovascular disease and cancer prevalence between Mediterranean and north European middle-aged populations (the Cilento on Ageing Outcomes Study and the Malmö Offspring Study). Intern Emerg. Med..

[61] Ohno Y., Iguchi A., Ijima M., Yasumoto K., Suzuki A. (2022). Coastal ecological impacts from pumice rafts. Sci. Rep..

[62] Willcox B., Willcox D.C., Suzuki M. (2004). The Okinawa Diet Plan. A Division of Random House.

[63] Willcox B.J., Willcox D.C., Todoriki H., Fujiyoshi A., Yano K., He Q., Curb J.D., Suzuki M. (2007). Caloric restriction, the traditional Okinawan diet, and healthy aging: The diet of the world’s longest-lived people and its potential impact on morbidity and life span. Ann. N. Y. Acad. Sci..

[64] Willcox D.C., Scapagnini G., Willcox B.J. (2014). Healthy aging diets other than the Mediterranean: A focus on the Okinawa Diet. Mech. Ageing Dev..

[65] Buettner D. (2009). The Blue Zones: Lessons for Living Longer from the People Who’ve Lived the Longest.

[66] Willcox D.C., Willcox B.J., Todoriki H., Suzuki M. (2009). The Okinawan diet: Health implications of a low-calorie, nutrient-dense, antioxidant-rich dietary pattern low in glycemic load. J. Am. Coll. Nutr..

[67] Sho H. (2001). History and characteristics of Okinawan longevity food. Asia Pac. J. Clin. Nutr..

[68] Colman R.J., Anderson R.M. (2011). Non human primate calorie restriction. Antioxid. Redox. Signal..

[69] Fontana L., Partridge L., Longo V.D. (2010). Extending healthy life span—From yeast to humans. Science.

[70] McCay C.M., Crowel M.F. (1934). Prolonging the life span. Sci. Mon..

[71] Suzuki M. (2001). Cultural climate and social custom for longevity region, Okinawa. Nihon Ronen Igakkai Zasshi.

[72] Hori M., Shozugawa K., Sugimori K., Watanabe Y. (2021). A survey of monitoring tap water hardness in Japan and its distribution patterns. Sci. Rep..

[73] Catling L.A., Abubakar I., Lake I.R., Swift L., Hunter P.R. (2008). A systematic review of analytical observational studies investigating the association between cardiovascular disease and drinking water hardness. J. Water Health.

[74] ISTAT (2023). Popolazione Residente 2023.

[75] United States Census Bureau An Official Website of the United States Government. https://www2.census.gov/programs-surveys/popest/tables/.

[76] Wang C., Murgia M.A., Baptista J., Marcone M.F. (2022). Sardinian dietary analysis for longevity: A review of the literature. J. Ethn. Foods.

[77] Rosero-Bixby L., Dow W.H., Rehkopf D.H. (2013). The Nicoya region of Costa Rica: A high longevity island for elderly males. Vienna Yearb. Popul. Res..

[78] HNMS Hellenic National Meteorological Service. Meteorological Observatory: 2023–2024. http://emy.gr/emy/en.

[79] Ungvari Z., Fazekas-Pongor V., Csiszar A., Kunutsor S.K. (2023). The multifaceted benefits of walking for healthy aging: From Blue Zones to molecular mechanisms. Geroscience.

[80] Loma Linda Climate Climate-Data.org—United States of America—California—Loma Linda. https://en.climate-data.org/north-america/united-states-of-america/california/loma-linda-15062/.

[81] Buettner D., Skemp S. (2016). Blue Zones: Lessons from the World’s Longest Lived. Am. J. Lifestyle Med..

[82] Lee J.W., Morton K.R., Walters J., Bellinger D.L., Butler T.L., Wilson C., Walsh E., Ellison C.G., McKenzie M.M., Fraser G.E. (2009). Cohort profile: The biopsychosocial religion and health study (BRHS). Int. J. Epidemiol..

[83] Seeman T.E. (1996). Social ties and health: The benefits of social integration. Ann. Epidemiol..

[84] Morton K.R., Lee J.W., Martin L.R. (2017). Pathways from Religion to Health: Mediation by Psychosocial and Lifestyle Mechanisms. Psychol. Relig. Spirit..

[85] Poulain M., Pes G., Salaris L. (2011). A population where men live as long as women: Villagrande Strisaili Sardinia. J. Aging Res..

[86] Pes G.M., Poulain M., Pachana N.A. (2016). Blue zones. Encyclopedia of Geropsychology.

[87] Pes G.M., Errigo A., Tedde P., Dore M.P. (2020). Sociodemographic, clinical and functional profile of nonagenarians from two areas of Sardinia characterized by distinct longevity levels. Rejuvenation Res..

[88] Fastame M.C., Ruiu M., Mulas I. (2021). Mental health and religiosity in the Sardinian blue zone: Life satisfaction and optimism for aging well. J. Relig. Health.

[89] Pes G.M., Tolu F., Dore M.P., Sechi G.P., Errigo A., Canelada A., Poulain M. (2015). Male longevity in Sardinia, a review of historical sources supporting a causal link with dietary factors. Eur. J. Clin. Nutr..

[90] Maioli M., Pes G.M., Sanna M., Cherchi S., Dettori M., Manca E., Farris G.A. (2008). Sourdough-leavened bread improves postprandial glucose and insulin plasma levels in subjects with impaired glucose tolerance. Acta Diabetol..

[91] Fastame M.C. (2022). Well-being, food habits, and lifestyle for longevity. Preliminary evidence from the sardinian centenarians and long-lived people of the Blue Zone. Psychol. Health Med..

[92] Passeri G., Pini G., Troiano L., Vescovini R., Sansoni P., Passeri M., Gueresi P., Delsignore R., Pedrazzoni M., Franceschi C. (2003). Low vitamin D status, high bone turnover, and bone fractures in centenarians. J. Clin. Endocrinol. Metab..

[93] Fondazione Valter Longo Proprietà e Fonti di Vitamina D. https://www.fondazionevalterlongo.org/proprieta-e-fonti-di-vitamina-d/#:~:text=Cereali%20integrali%2C%20frutta%20secca%20(mandorle,cavolo%20nero)%20contengono%20vitamina%20D.

[94] Blusztajn J.K., Slack B.E., Mellott T.J. (2017). Neuroprotective Actions of Dietary Choline. Nutrients.

[95] Corder R., Mullen W., Khan N.Q., Marks S.C., Wood E.G., Carrier M.J., Crozier A. (2006). Oenology: Red wine procyanidins and vascular health. Nature.

[96] Biasi F., Guina T., Maina M., Cabboi B., Deiana M., Tuberoso C.I., Calfapietra S., Chiarpotto E., Sottero B., Gamba P. (2013). Phenolic compounds present in sardinian wine extracts protect against the production of inflammatory cytokines induced by oxysterols in CaCo-2 human enterocyte-like cells. Biochem. Pharmacol..

[97] Ndlovu T., van Jaarsveld F., Caleb O.J. (2019). French and Mediterranean-style diets: Contradictions, misconceptions and scientific facts—A review. Food Res. Int..

[98] Ranabhat C.L., Park M.B., Kim C.B. (2020). Influence of alcohol and red meat consumption on life expectancy: Results of 164 countries from 1992 to 2013. Nutrients.

[99] Panagiotakos D.B., Chrysohoou C., Siasos G., Zisimos K., Skoumas J., Pitsavos C., Stefanadis C. (2011). Sociodemographic and lifestyle statistics of oldest old people (&80 years) living in Ikaria Island: The Ikaria study. Cardiol. Res. Pract..

[100] Legrand R., Manckoundia P., Nuemi G., Poulain M. (2019). Assessment of the health status of the oldest olds living on the Greek Island of Ikaria: A population-based study in a blue zone. Curr. Gerontol. Geriatr. Res..

[101] Institute of Environmental Research and Sustainable Development (IERSD) National Observatory of Athens: Athens, Greece. https://www.cleanenergywire.org/experts/institute-environmental-research-and-sustainable-development-iersd-greece.

[102] IMFE Institute of Mediterranean Forest Ecosystems. Institute of Research of Greece. Athens, Greece. https://www.lifeprimed.eu/en/institute-of-mediterranean-forest-ecosystems-hellenic-agricultural-organization-demeter.

[103] Pietri P., Papaioannou T., Stefanadis C. (2017). Environment: An old clue to the secret of longevity. Nature.

[104] Kritikou E., Kalogiouri N.P., Kostakis M., Kanakis D.C., Martakos I., Lazarou C., Pentogennis M., Thomaidis N.S. (2021). Geographical characterization of olive oils from the North Aegean region based on the analysis of biophenols with UHPLC-QTOF-MS. Foods.

[105] Zhang W. (2008). Religious participation and mortality risk among the oldest old in China. J. Gerontol. Ser. B.

[106] Tosi J.A.T.S. Center. Mapa Ecológico de Costa Rica. San José, Costa Rica: 1969. In collab. with J. Tosi. https://nla.gov.au/nla.obj-2568086976/view.

[107] Rosero-Bixby L. (2008). The exceptionally high life expectancy of Costa Rican nonagenarians. Demography.

[108] Rehkopf D.H., Dow W.H., Rosero-Bixby L. (2010). Differences in the association of cardiovascular risk factors with education: A comparison of Costa Rica (CRELES) and the USA (NHANES). J. Epidemiol. Community Health.

[109] Rosero-Bixby L., Dow W.H. (2012). Predicting mortality with biomarkers: A population-based prospective cohort study for elderly Costa Ricans. Popul. Health Metr..

[110] Momi-Chacòn A., Capitan-Jimenez C., Campos H. (2017). Dietary habits and lifestyle among long-lived residents from the Nicoya peninsula of Costa Rica. Rev. Hispanoam. Cienc. Salud..

[111] Mora-Alvarado D.M., Barquero C.F.P., Herrera N.A., Miraulth M.H. (2015). Diferencias de dureza del agua y las tasas de longevidad en la península de Nicoya y los otros distritos de Guanacaste. Tecnol. En Marcha.

[112] Mora-Alvarado D.M., Herrera N.A., Portuquez C.F., Brolatto M.P. (2000). Calculos en las vías urinarias y su relaciòn con el consumo de calcio en el agua de bebida en Costa Rica. Costa Rican J. Public Health.

[113] Chapman A.M. (1960). Los Nicarao y los Chorotega Según las Fuentes Históricas.

[114] Charlemagne-Badal S.J., Lee J.W. (2016). Intrinsic Religiosity and Hypertension among Older North American Seventh Day Adventists. J. Relig. Health.

[115] Fraser G.E., Shavlik D.J. (2001). Ten years of life: Is it a matter of choice?. Arch. Intern. Med..

[116] Fraser G.E. (2003). Diet, Life Expectancy, and Chronic Disease: Studies of Seventh Day Adventists and Other Vegetarians.

[117] Weaver A.J., Pargament K.I., Flannelly K.J., Oppenheimer J.E. (2006). Trends in the scientific study of religion, spirituality, and health: 1965–2000. J. Relig. Health.

[118] McCullough M.E., Hoyt W.T., Larson D.B., Koenig H.G., Thoresen C. (2000). Religious involvement and mortality: A meta-analytic review. Health Psychol..

[119] Powell L.H., Shahabi L., Thoresen C.E. (2003). Religion and spirituality. Linkages to physical health. Am. Psychol..

[120] Seybold K.S., Hill P.C. (2001). The role of religion and spirituality in mental and physical health. Curr. Dir. Psychol. Sci..

[121] Hall D.E. (2006). Religious attendance: More cost-effective than Lipitor?. J. Am. Board Fam. Med..

[122] Hummer R.A., Ellison C.G., Rogers R.G., Moulton B.E., Romero R.R. (2004). Religious involvement and adult mortality in the United States: Review and perspective. South Med. J..

[123] Muraki I., Rimm E.B., Willett W.C., Manson J.E., Hu F.B., Sun Q. (2016). Potato consumption and risk of type 2 diabetes: Results from three prospective cohort studies. Diabetes Care.

[124] Nieddu A., Vindas L., Errigo A., Vindas J., Pes G.M., Dore M.P. (2020). Dietary Habits, Anthropometric Features and Daily Performance in Two Independent Long-Lived Populations from *Nicoya peninsula* (Costa Rica) and *Ogliastra* (Sardinia). Nutrients.

[125] Aliberti S.M., Corposanto C. (2017). Le badanti e la cura domiciliare: Come tassello dei servizi integrati del welfare locale. Narrazioni di Salute Nella Web Society.

[126] United Nations Sustainable Development Goals. Take Action for the Sustainable Development Goals. https://www.un.org/sustainabledevelopment/sustainable-development-goals/.

[127] Convention on Biological Diversity Strategic Plan for Biodiversity 2011–2020, Including Aichi Biodiversity Targets. https://www.cbd.int/sp.

[128] Fara G.M. (2015). Nutrition between sustainability and quality. Ann. Ig..

[129] Ma E., Ohira T., Yasumura S., Hosoya M., Miyazaki M., Okazaki K., Nagao M., Hayashi F., Nakano H., Eguchi E. (2022). Development of a Japanese Healthy Diet Index: The Fukushima Health Management Survey 2011. Int. J. Environ. Res. Public Health.

[130] Dalghren G., Whitehead M. (2007). Policies and Strategies to Promote Social Equity in Health.

